# Treatment of Lupus Nephritis from Iranian Traditional Medicine and Modern Medicine Points of View: A Comparative Study

**DOI:** 10.1155/2021/6645319

**Published:** 2021-11-09

**Authors:** Yasaman Vahedi-Mazdabadi, Mina Saeedi

**Affiliations:** ^1^Persian Medicine and Pharmacy Research Center, Tehran University of Medical Sciences, Tehran, Iran; ^2^Medicinal Plants Research Center, Faculty of Pharmacy, Tehran University of Medical Sciences, Tehran, Iran

## Abstract

**Objective:**

Nephritis or kidney inflammation is characterized as one of the most common renal disorders leading to serious damage to the kidneys. Nephritis, especially lupus nephritis (LN), has remained as the main cause of chronic renal failure which needs serious therapeutic approaches such as dialysis and kidney transplant. Heredity, infection, high blood pressure, inflammatory diseases such as lupus erythematosus and inflammatory bowel disease, and drug-related side effects are known as the main causes of the disease. According to Iranian traditional medicine (ITM), infectious diseases and fever are the main reasons of nephritis, which is called “Varam-e-Kolye” (VK).

**Results:**

There are various plant-based remedies recommended by ITM for the treatment of nephritis, as discussed herein, comparing with those available in the modern medicine. There is no definite cure for the treatment of nephritis, and immunosuppressive drugs such as corticosteroids and nonsteroidal anti-inflammatory drugs, antibiotics, diuretics, analgesics, and finally dialysis and kidney transplantation are usually used. Based on the efficacy of medicinal plants, jujube (*Ziziphus jujuba*), almond (*Prunus amygdalus*), pumpkin seeds (*Cucurbita pepo*), purslane (*Portulaca oleracea*), and fig (*Ficus carica*) were found to be effective for the treatment of kidney inflammation in ITM.

**Conclusion:**

Considering the fact that there is no efficient strategy for the treatment of nephritis, use of herbal medicine, particularly based on the fruits or nuts that have been safely used for several years can be considered as a versatile supplement along with other therapeutic methods.

## 1. Introduction

Nephritis is characterized as the inflammation of kidney, affecting the glomerulus, tubule, or interstitial renal tissues [1, 2]. Although lots of therapeutic approaches have been recently developed for the treatment of various diseases, nephritis has still remained as the main cause of chronic kidney failure leading to significant financial burden on the societies [3]. Nephritis was ranked as 17th principal cause of death in 2002. Moreover, the number of cases of nephritis is increasing and it is expected to be the 13th leading cause of death in 2030 [4]. Various factors such as heredity including APOL1 gene variations [5], infection [6], hypertension [7], inflammatory diseases such as systemic lupus erythematosus (SLE) [8] and inflammatory bowel disease [9], drug-related side effects, and unknown causes [10] have been involved in the pathogenesis of the disease. Among these factors, SLE, simply called lupus, is one of the most significant causes of nephritis, which is known as lupus nephritis (LN). In fact, sedimentation of inflammatory mediators in the kidney tissue as the main result of SLE leads to glomerulonephritis and tubulointerstitial inflammation [11, 12].

Various studies have confirmed that the activation of nuclear factor kappa B (NF-*κ*B) signal pathway *via* upregulation of proinflammatory mediators such as cyclooxygenase-2 (COX-2) and tumor necrosis factor-*α* (TNF-*α*) is responsible for LN [13]. Also, it has been demonstrated that B- and T-cell hyperactivity [14], increased T helper 1 (Th1) and T helper 17 (Th17) cytokines [15, 16], activation of JAK/STAT-3 signaling [17], elevation of anti-double-stranded DNA (anti-dsDNA) antibodies [12], autoantibodies against proteins involved in clearing extracellular nuclear material such as pentraxin 3 or C-reactive protein [18], interleukin-6 (IL-6), interleukin-17 (IL-17), interleukin-23 (IL-23), TNF-*α* [19, 20], oxidative stress [14], decreased T regulator cells (Tregs) [21], and complement system [22] play important roles in the pathophysiology of LN.

LN is currently treated with an immunosuppressive regimen including induction therapy and then a maintenance immunosuppression phase [8, 18]. However, despite using aggressive therapies, the outcome and prognosis of LN have not been resolved since the 1980s [8] and up to 50% of patients encounter organ damage and end-stage renal disease (ESRD) [8, 18]. Some of these difficulties are indirectly associated with side effects of therapy [23, 24]. Furthermore, immunosuppressive drugs have shown severe adverse effects and a great number of patients have been prohibited to consume them [25]. Although novel approaches including anti-CD20 (B-lymphocyte antigen CD20) antibodies and calcineurin inhibitors have shown encouraging results, their long-term benefit/risk ratios have not been specified [26]. Hence, it would be desirable to provide treatments based on societal dietary and food with no side effects [27]. In fact, some of the foodstuffs can be used as functional food in the treatment of this disease [28].

Traditional medicine, profiting from medicinal plants and dietary strategies, has played a significant role in the treatment of different diseases such as kidney diseases [29–31]. In ITM, nephritis has been known as “Varam-e-Kolye” (VK) [32]. It is believed that infectious diseases, fever, strike, severe pressure on the kidney, urinary retention, scratch caused by kidney stones, increased blood volume, and untreated disease background are the main causes of nephritis or kidney swelling [32–34]. ITM has recommended safe herbal remedies for the treatment nephritis, most of which can be used in everyday life [31–34]. However, it is worth mentioning that some plants need a special attention to avoid severe problems such as toxicity [35–37].

In this review, treatment of nephritis has been discussed from the ITM point of view comparing with modern medicine, and moreover, some renal adverse effects related to medicinal plants were discussed.

## 2. Methods

Herein, credible Iranian traditional medicine resources such as “*Qanun-fil-Tibb*'“ (the Law of Medicine), “*Exir-e-Azam*” (Great Elixir), “*Makhzan-al-Advieh*” (the Storehouse of Medicaments), and “*Tibb-e-Akbari*” (Akbar's medicine) were studied using keywords including “varam” and “kolye”. Also, recent developments were reviewed in modern medicine using search engines such as Google Scholar, PubMed, Scopus, SID, and Iranmedex using keywords such as “inflammation + kidney,” “nephritis,” “lupus nephritis,” “systemic lupus erythematosus,” “medicinal plant,” “nephropathy,” “nephrotoxicity,” “hyperkalemia,” “hyperphosphatemia,” “plant-based diets,” “NF-kB,” “nutraceutical,” and “treatment” without time or language restrictions. Materials related to study criteria were extracted and analyzed in a comprehensive manner.

## 3. Results

According to the World Health Organization (WHO) or the International Society of Nephrology/Renal Pathology Society (ISN/RPS) class III and upper advances to ESRD, LN or kidney inflammation induced by SLE is described as one of the most common renal diseases [38–40]. Although the disease affects all parts of the kidney, the clinical evidence indicated that the glomerulus has the highest amounts of deposition of immune complexes and is involved in inflammation and formation of lesions [40–42] (Table 1).

At present, there is no definite cure for the treatment of nephritis in modern medicine [8], and usually, immunosuppressive drugs such as corticosteroids [8, 25] and nonsteroidal anti-inflammatory [25], renin-angiotensin-aldosterone system (RAAS) blockade [41], antibiotics [40], diuretics [43], dialysis [38, 44], and kidney transplantation [45] are recommended. However, in the early stages of LN including class I and II, no therapy is recommended by the American College of Rheumatology (ACR) guideline. The European LeagueAgainst Rheumatism and European Renal Association-European Dialysis and Transplant Association (EULAR/ERA-EDTA) suggested low to moderate doses of oral steroid agents which cause moderate proteinuria and glomerular hematuria [18, 41, 46, 47]. It should be noted that 5–20% patients with LN even with immunosuppressive therapy develop ESRD within 10 years [43]. Therefore, developing an efficient therapy for the treatment of inflammation of kidneys is highly in demand. In this regard, herbal remedies recommended by ITM have attracted lots of attention as the versatile strategy for the treatment of inflammation of kidneys (Table 2). However, some adverse effects demonstrated by some plants should not be forgotten (Table 3) [2, 83]. Also, special attention should be paid to the probable interaction of some herbs and different medications used by patients [83, 90–93]. Some plants have a significant amount of potassium, which can cause hyperkalemia (potassium > 5.5 mmol/l) in patients. Hence, the potassium content of these plants should also be considered [83, 94, 95] (Table 4).

### 3.1. Plants with Renoprotective Effects

#### 3.1.1. *Anethum graveolens*

*A. graveolens*, known as sheviid in ITM, has been widely utilized to reduce renal swelling [32, 33]. Khalil et al. indicated that oral ingestion of alcoholic extract of *A. graveolens* seeds in diabetic treated animals by alloxan diminished serum urea and creatinine and increased the serum glutathione concentration [48]. Kim et al. showed that the methanolic extract of *A. graveolens* flowers suppressed inducible nitric oxide synthase (iNOS) and inhibited NF-*κ*B activity *via* inhibiting the phosphorylation of serine/threonine kinase 1 (Akt) in lipopolysaccharide (LPS)-stimulated RAW 264.7 cells. Thus, it has an anti-inflammatory effect on LPS-stimulated RAW 264.7 cells [49].

#### 3.1.2. *Carum carvi*

*C. carvi* seeds, known as zeerah-siyah in ITM, have been used to treat kidney swelling [33]. Sadiq et al. reported that the aqueous extract of *C. carvi* seeds exhibited renoprotective effects against streptozotocin-induced nephropathy in rats [50]. Likewise, in another study conducted by El-Soud et al., the renoprotective effect of *C. carvi* essential oil in streptozotocin-induced diabetic rats was examined. The results represented that the bioactive compounds of *C. carvi* exhibited renoprotection by increasing the activity of the antioxidant enzymes [51]. Moreover, Lahlou et al. demonstrated that the aqueous extract of *C. carvi* induced furosemide-like diuretic effects in rats [52].

#### 3.1.3. *Coriandrum sativum*

*C. sativum*, known as geshniz in ITM, has been frequently used for the treatment of nephritis [32–34]. Lakhera et al. confirmed the nephroprotective role of the ethyl acetate extract of *C. sativum* in albino Wistar rats. It was illustrated that the extract significantly decreased creatinine levels, serum urea, and blood urea nitrogen. Furthermore, it improved renal histological lesions [53]. In another study, the renoprotective effect of combined administration of *Cuminum cyminum* and *Coriandrum sativum* in albino mice was proven by Kumar et al. [54]. Also, in a study conducted by El-Masry et al., *C. sativum* in combination with *Foeniculum vulgare* showed a significant improvement of serum creatinine and uric acid levels and boosted enzymatic and nonenzymatic antioxidants levels compared with the lead group [55]. Wu et al. proved that the ethanolic extracts of leaves and stems of *C. sativum* inhibited NF-*κ*B activity and the mitogen-activated protein kinase (MAPK) signal transduction pathway in LPS-induced macrophages and had an anti-inflammatory effect [56].

#### 3.1.4. *Cucurbita pepo*

*C. pepo* seeds, known as tokhme-kadoo in ITM, have been broadly expended in Iran as a nephroprotective agent [33]. Adepoju and Adebanjo confirmed the nephroprotective activity of *C. pepo* seeds in albino rats [57].

#### 3.1.5. *Cydonia oblonga*

*C. oblonga*, known as behi in ITM, is another plant used for the treatment of nephritis in Iran [32–34]. Mirmohammadlu et al. depicted that the aqueous extract of *C. oblonga* improved renal function in streptozotocin-induced diabetic rats. Accordingly, the extract decreased serum urea and creatinine levels [58]. Also, Aggarwal and Shishodia indicated the effect of caffeoylquinic acids obtained from *C. oblonga* on the reduction of NF-*κ*B and other inflammatory factors such as interferon gamma (IFN-*γ*), interleukin-2 (IL-2), extracellular signal-regulated kinases (ERK1/2), AKTa, nitric oxide (NO), and iNOS [59].

#### 3.1.6. *Ficus carica*

The fruits of *F. carica*, known as anjir in ITM, have been recommended as a versatile remedy for nephritis [32, 33]. As reported by Kore et al., the hydroalcoholic extract of *F. carica* ameliorated gentamicin-induced nephrotoxicity in rats [60]. The extract enhanced catalase (CAT) and glutathione (GSH) concentrations and decreased the level of malondialdehyde (MDA) [60]. In a study conducted by Sharma et al., oral administration of morin derived from *F. carica* to male albino Wistar rats suppressed the NF-*κ*B pathway and lessened inflammatory mediators such as TNF-*α*, IL-6, COX-2, and prostaglandin (PGE-2) [61].

#### 3.1.7. *Linum usitatissimum*

*L. usitatissimum* seeds, known as bazr-e-katan in ITM, have been extensively consumed to reduce kidney inflammation [32, 33]. The nephroprotective effect of ground flaxseed in patients with lupus nephritis was assessed by Clark et al. [62]. In another study by Akpolat et al., the protective effect of seeds of *L. usitatissimum* on renal injury in rats was investigated. The results illustrated a decreased deposition of neutral lipid. Moreover, flaxseed alleviated renal injuries associated with hypercholesterolemia [63].

#### 3.1.8. *Melissa officinalis*

*Melissa officinalis* L., known as badranjboya in ITM, has been frequently applied as a remedy for kidney inflammation [33, 96]. Nephroprotective activity and anti-inflammatory effects of *M. officinalis* leaf extract against acetaminophen- and pleurisy-induced toxicity in rats were studied by Müzell et al. It was revealed that the aqueous extract possessed nephroprotective effects, leading to a decrease in the inflammation of kidney [64]. As reported by Hamza et al., oral administration of the ethanolic extract of *M. officinalis* leaves to male albino rats inhibited inflammatory responses to doxorubicin (DOX)-induced inflammation by reducing the expressions of NF-*κ*B, TNF-*α*, and COX-2 [65].

#### 3.1.9. *Prunus amygdalus*

*P. amygdalus* kernels, known as badam in ITM, have been frequently prescribed to improve the inflammation of kidney [32–34]. Renoprotective effects of hexane-isopropyl alcohol extract of almond kernels in streptozotocin-induced diabetic rats were reported by Demir et al. The extract reduced the MDA levels and GSH in the renal tissue [66]. Also, Pandey et al. proved the renal protective effect of ethanolic extract of *P. amygdalus* seeds in male Swiss albino rats. The results indicated that using the extract in a dose-dependent manner detracted the IL-6, IL-1b, TNF-*α*, and inflammatory mediators PGE-2 and NF-*κ*B. Also, the renoprotective effect of *P. amygdalus* was observed in histopathological analyses [67]. Jamshed et al. showed that daily consumption of almond supplementation (10 mg) before breakfast diminished serum uric acid in coronary artery disease patients [68].

#### 3.1.10. *Ziziphus jujuba* Mill

*Z. jujuba* fruits, known as onab in ITM, have been used to treat nephritis [34]. Awad et al. investigated the renoprotective activity of the aqueous extract of *Z. jujuba* in rats by inducing nephrotoxicity with ibuprofen. The results demonstrated a significant decrease in serum urea and creatinine. This study suggested that taking ibuprofen with aqueous *Z. jujuba* extracts prevented the side effects of ibuprofen [69]. In the study reported by Zhao et al., the effect of injection of a patent herbal drug decoction prepared from 16 herbs including *Z. jujuba*, on the rat model of chronic glomerulonephritis, was evaluated. The results exhibited that the herbal drug decoction downregulated the proteins levels of transforming growth factor beta 1 (TGF-*β*1), phospho-small mothers against decapentaplegic (Smad)2/3 (Thr8), and Smad4 in rat renal tissues. It also inhibited the TGF-*β*/Smad signaling pathway and improved all abnormal behavioral and biochemical changes in the model rats [70] (Table 2).

### 3.2. Plants with Adverse Renal Effects

Plants can cause side effects due to the presence of secondary metabolites and active compounds. Although they have shown beneficial effects in the treatment of kidney disease, some have demonstrated harmful effects [97]. The kidneys are susceptible to a wide variety of toxic insults due to high concentration of these compounds in the medullary interstitium and active uptake by tubular cells [83]. Adverse effects of herbal medicines on the kidneys can appear in different forms such as nephritis, hypertension, acute tubular necrosis, acute interstitial nephritis, Fanconi's syndrome, papillary necrosis, chronic interstitial renal fibrosis, urinary retention, kidney stones (nephrolithiasis, renal lithiasis), urinary tract carcinoma, hemorrhagic complications, and rhabdomyolysis [83]. Also, it can lead to food-drug interactions and changes in the potassium level [98, 99]. Herein, some reported adverse effects have been listed in Table 3.

#### 3.2.1. Plants with Nephrotoxicity Effects

*(1) Aloe ferox*. The *A. ferox leaves*, known as Sabr-e-zard in ITM [96], contain aloesin (or aloeresin B) and aloeresin A [71] that have shown acute oliguric renal failure [71], interstitial nephritis, and anuria [71].

*(2) Aristolochia species. Aristolochia* species roots, known as zaravand in ITM [96], include aristolochic acid (AA) [74] that causes chronic interstitial renal fibrosis [72], proximal tubular toxicity [73], aristolochic acid nephropathy (AAN) [74, 75], severe hypocellular interstitial fibrosis, urothelial dysplasia [76], renal failure with interstitial fibrosis [77], Balkan endemic nephropathy (BEN) [78, 79], and Fanconi's syndrome [73, 80–82]. It seems that tubular compartment is the main target of AA and AA exerts apoptotic effects *via* activating mitochondrial permeability and activating caspase-3 [100]. Moreover, other mechanisms, including blocking out regeneration by changing epidermal growth factor (EGF) and vascular endothelial growth factor (VEGF) expression; increasing of epithelial-mesenchymal transition resulting in the accumulation of vimentin and *α*-smooth muscle actin-positive cellsprobably *via* the influence of TGF-*β* and/or connective tissue growth factor (CTGF); and elevating the production of extracellular matrix components in the interstitium, have been reported in the literature [100–104]. Also, this compound is carcinogenic and uroepithelial cancers have been indicated in about 40–46% of patients who received total doses of AA higher than 200 g [74, 105, 106]. The carcinogenesis of AA is related to the strong affinity of AA metabolites for the exocyclic amino group of DNA and after binding to the adenine residues; these metabolites induce activation of Harvey rat sarcoma viral oncogene homolog (H-ras) and overexpression of tumor protein P53 (p53) [100, 107, 108].

*(3) Ephedra sinica*. The branches and fruits of *E. sinica*, known as houm or rish-e-boz in ITM [96], contain ephedrine [83] that can lead to hypertension [83], formation of kidney stones [84], nephritic colic [84], and rhabdomyolysis [85].

*(4) Glycyrrhiza glabra. G. glabra*. roots, known as shirin bayan in ITM [96], contain glycyrrhizic and glycyrrhetinic acids [109] that possess mineralocorticoid activity and can cause hypokalemia, sodium and water retention, metabolic alkalosis, hypertension, and suppression of the renin-aldosterone system [86]. Also, Fanconi's tubulopathy [87], proximal tubulopathy [88], and hypokalemic rhabdomyolysis along with acute renal failure [89] have been reported by the consumption of *G. glabra*.

#### 3.2.2. Plants Interactions with Conventional Drugs

Some plants have metabolites that can provoke or inhibit the cytochrome P450 3A4 (P450 3A4) enzyme, which is the main enzyme involved in drug metabolism, and the drug transport protein P-glycoprotein 1 (p-gp) [2]. In this respect, they accelerate the clearance of drugs and diminish cyclosporine or tacrolimus bioavailability [2]. Also, these plants can raise the blood concentration of prednisolone [97]. Another interaction relates to the significant increase in the metabolism of immunosuppressive drugs, which causes the blood concentrations of those drugs to be reduced lower than the therapeutic levels, and the organ transplant recipients are prone to transplant rejection due to lower cyclosporine blood concentrations [110, 111].

One of the important plants that reduces the blood levels of cyclosporine is *Hypericum perforatum* that is known as St John's wort. One of the metabolites of St John's wort is hyperforin, which is a potent ligand for a nuclear receptor that regulates the expression of P450 3A4 monooxygenase [91, 93, 112].

#### 3.2.3. Plants Induce Changes in the Potassium Level

Some plants contain considerable amounts of potassium that may lead to hyperkalemia, chiefly in patients with kidney problems including chronic renal failure (Table 4) [83, 98]. Renal patients, who are particularly prone to hypokalemia and hyperkalemia, need dietary potassium restriction since intake of high potassium dietary levels can lead to the accumulation of potassium in patients with reduced kidney function and hyperkalemia [113, 114]. However, the drugs such as cyclosporine and tacrolimus induce hyperkalemia *via* inhibition of adrenal aldosterone biosynthesis [113]. Hyperkalemia can lead to metabolic acidosis and malignant arrhythmias as well as higher mortality [95].

Some potassium-rich plants such as alfalfa (*Medicago sativa*) and dandelion (*Taraxacum officinale*) can cause hyperkalemia particularly in the chronic renal failure patients. However, some plants, even with a low content of potassium, can lead to hyperkalemia, e.g., noni juice (*Morinda officinalis*) with potassium content of 56.3 mEq/L [83, 115].

## 4. Discussions

Currently, immunosuppressive drugs are the most common drugs in the treatment of nephritis. However, incidence of different complications, in around 1/3 of patients [25] using them, has led to the development of novel therapeutic strategies. In this respect, herbal remedies have attracted lots of attention due to their NF-*κ*B inhibitory potency through binding to peroxisome proliferator-activated receptor (PPAR)*γ* [116, 117]. Considering the fact that NF-*κ*B is one of the most important proinflammatory cytokines causing nephritis, plant-based treatment can be a strong tool in the treatment of LN. ITM, which has focused on the properties of medicinal herbs, has provided useful remedies for LN. Most of these plants have been daily consumed for their culinary and medicinal goals for hundreds of years [118], and their therapeutic effects for the kidney disease and nephritis have been proven in various studies [119].

Among the plants recommended by ITM for the treatment of nephritis, some, including badranjboya (*M. officinalis*) [65], sheviid (*A. graveolens*) [49], geshniz (*C. sativum*) [56], behi (*C. oblonga*) [59], anjir (*F. carica*) [61], and badam (*P. amygdalus*) [67] have shown NF-*κ*B inhibitory activity. Moreover, many of these plants have also diminished other inflammatory factors such as iNOS, IL-6, TNF-*α*, and COX-2 [49, 59, 61, 65].

Various studies have shown that vitamin deficiencies can cause LN and exacerbate inflammation in patients with SLE and kidney disease [120, 121]. Plants that are rich in vitamin A, vitamin E, vitamin D, vitamin C, and vitamin B6 have suppressed anti-dsDNA autoantibody production, Th17 cells, interleukin-17A (IL-17A)-producing T cells, lipid peroxidation, active inflammation, and IL-6 secretion and increased Treg percentages in CD4+ T cells in patients with SLE [28, 122]. Also, a number of studies have presented the favorable effects of these vitamins that decreased IL-2, IFN-*γ*, and interleukin-12 (IL-12) and interleukin-4 (IL-4) in serum as well as immunoglobulin G2 (IgG2)-specific anti-DNA antibody. They also increased TGF-*β* and forkhead box P3 (FOXP3) mRNA expression and suppressed iNOS and monocyte chemoattractant protein-1 (MCP-1) in the kidney in the SLE models [28].

Plant food sources in addition to their role in providing dietary macronutrients and micronutrients are the major dietary sources of different plant secondary metabolites such as polyphenolic compounds including flavones, flavonols, flavan-3-ols, isoflavones, flavanones, and anthocyanidins, which play beneficial roles in human health [28, 123]. Different studies in patients with SLE and SLE models have revealed that plant secondary metabolites and polyphenolic compounds play an important role in the improvement of inflammatory processes in SLE leading to LN and renal complications, by suppressing IFN-*γ* response and anti-dsDNA; reducing serum antinuclear antibodies and cytokines IL-17A, IL-1*β*, TNF-*α*, and IL-6; decreasing reactive oxygen species (ROS) levels in serum; decreasing serum creatinine and microalbumin; improving renal function, plasma lipids, blood viscosity; increasing complement C3 levels; decreasing proteinuria and splenic lymphocyte proliferation; and attenuating glomerular filtration and lymphoproliferation [28, 121, 124].

Moreover, these plants including almond, flaxseed, pumpkin seeds, and caraway seeds are the source of n-6 polyunsaturated fatty acid (PUFA), n-3 PUFA, and monounsaturated fatty acid (MUFA) that have decreased fibronectin-1, intercellular adhesion molecule-1 (ICAM-1), TGF-*β*1 mRNA levels and TGF-*β*1 protein, IL-1*β*, IL-6, and TNF-*α* in the kidney; increased antioxidant enzymes such as catalase, glutathione peroxidase (GPx), and superoxide dismutase (SOD) in the kidney; reduced the erythrocyte sedimentation rate and serum IL-12 levels, reduced platelet arachidonic acid, neutrophil leukotriene B4, and TAG; reduced the expressions of CD80, cytotoxic T-lymphocyte-associated protein 4 (CTLA-4), IL-10, C-C motif chemokine ligand 5 (CCL-5), C-X-C motif chemokine receptor 3 (CXCR3), and osteopontin mRNA in the kidney; downregulated NF-*κ*B activation; decreased serum anti-dsDNA antibodies; ameliorated nuclear factor erythroid 2-related factor 2 (Nrf-2), heme oxygenase-1 (HO-1), JAK/STAT, MAPK, and NF-*κ*B pathways in the kidney; and decreased serum matrix metalloproteinase-3 (MMP-3) and PGE-2 in the kidney in SLE models and patients with SLE [28, 121].

Although plants have depicted versatile effects on the treatment of inflammatory diseases such as lupus nephritis, they need an important attention as they may contain high levels of sodium, potassium, and phosphorus, leading to hypertension, fluid retention, hyperkalemia, and hyperphosphatemia. Hyperkalemia and hyperphosphatemia directly raise patient mortality in advanced CKD [114, 125, 126]. A low estimated glomerular filtration rate (eGFR) restricts renal potassium and phosphorus excretion in patients with CKD especially in ESRD, causing hyperkalemia and hyperphosphatemia [114, 126]. Hypertension, fluid retention, hyperkalemia, and hyperphosphatemia can affect heart function, leading to acute myocardial ischemia, left ventricular hypertrophy, cardiac arrhythmia, congestive heart failure, and mortality [114, 125, 127, 128]. So, the patients with low eGFR are recommended to restrict dietary potassium, phosphorus, and plant-based diets such as seeds, nuts, fruits, and vegetables [125, 128].

However, recent studies have demonstrated that the bioavailability of potassium and phosphorus in plant-based diets is less than meat-based diets due to high fiber content found in plant food, which may lead to steady serum potassium and phosphorus levels, correct metabolic acidosis, delay advancement to dialysis and ESRD, and mitigate mortality [128, 129]. Furthermore, it has been revealed that restriction in alimentary potassium and phosphorus intake resulted in unplanned diminutions in beneficial macronutrients especially reducing protein, which is associated with the occurrence of malnutrition in patients' kidney. On the other hand, it was shown that potassium- and phosphorus-limiting diets have little effect on maintaining normal serum potassium and phosphorus levels [128–131]. The use of plant-based diets in CKD may have other advantages including decrease in weight, hypertension, hyperphosphatemia, hyperfiltration, and possibly mortality [132]. Furthermore, plant-based diets are beneficial as long as the diet is properly implemented to provide adequate protein intake, including essential amino acids [132].

Goldner studied the effect of raw, whole-food, plant-based (WfPB) diets on two patients with SLE-related nephritis. The results illustrated that in Case 1, the eGFR enhanced from 14 to 27 ml/min in 6 weeks and did not need dialysis or a kidney transplant. In Case 2, laboratory tests were normalized and symptoms were improved due to dietary changes to WfPB diets [133].

It should be noted that some plants such as *G. glabra*, *Aristolochia* species, *A. ferox*, and *E. sinica* have demonstrated nephrotoxicity effects and need the attention of patients who are affected by nephritis and kidney inflammation. Therefore, nutritional interventions in the renal patients should be considered for the possible renal toxicity provided by plants [2, 83].

## 5. Conclusion

ITM has provided a plant-based approach to nephritis to preserve or recover renal function. These plants have depicted anti-inflammatory effects *via* different pathways and are prone to be developed as dietary supplements.

## Figures and Tables

**Table 1:** International Society of Nephrology/Renal Pathology Society (ISN/RPS) 2003 classification of lupus nephritis [39].

Class I	Minimal mesangial LN
Class II	Mesangial proliferative LN
Class III	Focal LN (50% of glomeruli)
III (A)	Active lesions
III (A/C)	Active and chronic lesions
III (C)	Chronic lesions
Class IV	Diffuse LN (50% glomeruli)
	Diffuse segmental (IV-S) or global (IV-G) LN
IV (A)	Active lesions
IV (A/C)	Active and chronic lesions
IV (C)	Chronic lesions
Class V	Membranous LN
Class VI	Advanced sclerosing LN (90% globally sclerosed glomeruli without residual activity)

**Table 2:** Plants used in the treatment of lupus nephritis in Iranian traditional medicine.

Species	English name	Iranian name	Family	Parts used	Extract	Activity	Reference
*A. graveolens*	Dill	Sheviid	Apiaceae	Seeds	Alcoholic extract	Decrease in serum urea and creatinine and increase in serum glutathione concentration	[48, 49]

*C. carvi*	Caraway	Zeerah-siyah	Apiaceae	Seeds	Aqueous extract	Renoprotective	[50–52]

*C. sativum*	Coriander	Geshniz	Apiaceae	Leaves	Ethyl acetate	Nephroprotective potential suppressing NF-*κ*B activation and MAPK signal transduction pathway	[53–56]

*C. pepo*	Pumpkin	Kadoo	Cucurbitaceae	Seeds		Renoprotective	[57]

*C. oblonga*	Quince	Behi	Rosaceae	Fruits	Aqueous extract	Reducing serum urea and creatinine; reduction of IFN-g, IL-2, ERK1/2, AKTa, NF-*κ*B, NO, iNOS	[58, 59]

*F. carica*	Fig	Anjir	Moraceae	Fruits	Hydroalcoholic extract	Nephroprotective activity decreasing inflammatory mediators such as TNF-*α*, IL-6, COX-2, and PGE-2	[60, 61]

*L. usitatissimum*	Flaxseed	Bazr-e-katan	Linaceae	Seeds	Ground flaxseed, oil	Protective effect on renal injury	[62, 63]

*M. officinalis*	Lemon balm	Badranjboya	Lamiaceae	Leaves	Aqueous extract	Nephroprotective and anti-inflammatory effects reducing the expressions of NF-*κ*B, TNF-*α*, and COX-2	[64, 65]
					Ethanolic extract		

*P. amygdalus*	Almond	Badam	Rosaceae	Kernels	Hexane-isopropyl alcohol extract, ethanolic extract	Renoprotective effect, diminishing serum uric acid	[66–68]

*Z. jujuba Mill.*	Jujube	Onab	Rhamnaceae	Fruits	Aqueous extract injection, a patent herbal drug decoction	Reducing nephrotoxicity induced by ibuprofen, downregulated protein levels of TGF-*β*1, phospho-Smad2/3 (Thr8) and Smad4 in rat renal tissues, and inhibition of the TGF-*β*/Smad signaling pathway	[69, 70]

**Table 3:** Examples of herbal plants with nephrotoxicity effects.

Species	English name	Iranian name	Family	Risk	Reference
*Aloe ferox*	Cape aloe	Sabr-e-zard	Asphodelaceae	Acute oliguric renal failure, interstitial nephritis, anuria	[71]
					[71]

*Aristolochia species*	Birthwort	Zaravand	Aristolochiaceae	Interstitial renal fibrosis, proximal tubular toxicity, aristolochic acid nephropathy (AAN), severe hypocellular interstitial fibrosis, urothelial dysplasia, renal failure with interstitial fibrosis, Balkan endemic nephropathy (BEN), Fanconi's syndrome	[72]
					[73]
					[74–76]
					[77]
					[78, 79]
					[73, 80–82]

*Ephedra sinica*	Ephedra	Houm or rish-e-boz	Ephedraceae	Hypertension, formation of kidney stones, nephritic colic, rhabdomyolysis	[83]
					[84]
					[84]
					[85]

*Glycyrrhiza glabra*	Liquorice	Shirin bayan	Fabaceae	Renin-aldosterone system, Fanconi's tubulopathy, proximal tubulopathy, hypokalemic rhabdomyolysis along with acute renal failure	[86]
					[87]
					[88]
					[89]

**Table 4:** Fruit and vegetable sources of potassium, ranked by milligrams of potassium per standard amount^*∗*^.

Fruits and vegetables, standard amount	Potassium (mg)
Sweet potato, baked, 1 potato (146 g)	694
Tomato paste, ¼ cup	664
Beet greens, cooked, ½ cup	655
Potato, baked, flesh, 1 potato (156 g)	610
White beans, canned, ½ cup	595
Tomato puree, ½ cup	549
Prune juice, ¾ cup	530
Carrot juice, ¾ cup	517
*Lima* beans, cooked, ½ cup	484
Winter squash, cooked, ½ cup	448
Banana, 1 medium	422
Spinach, cooked, ½ cup	419
Tomato juice, ¾ cup	417
Tomato sauce, ½ cup	405
Peaches, dried, uncooked, ¼ cup	398
Prunes, stewed, ½ cup	398
Apricots, dried, uncooked, ¼ cup	378
Cantaloupe, ¼ medium	368
Honeydew melon, 1/8 medium	365
Plantains, cooked, ½ cup slices	358
Kidney beans, cooked, ½ cup	358
Orange juice, ¾ cup	355
Split peas, cooked, ½ cup	355

^*∗*^ US Department of Health and Human Services and US Department of Agriculture, 2005. The dietary reference intake (DRI) for potassium for adults and adolescents is 4700 mg/day.

## Data Availability

The data supporting this review are from the previously reported studies and datasets, which have been cited. The data used to support the findings of this study are available from the corresponding author upon request.

## Abbreviations

LN: Lupus nephritis
ITM: Iranian traditional medicine
VK: Varam-e-Kolye
SLE: Systemic lupus erythematosus
NF-*κ*B: Nuclear factor kappa B
COX-2: Cyclooxygenase-2
Th1: T helper 1
Th17: T helper 17
Anti-dsDNA: Anti-double-stranded DNA
IL-6: Interleukin-6
IL-17: Interleukin-17
IL-23: Interleukin-23
TNF-*α*: Tumor necrosis factor-*α*
Tregs: T regulator cells
ESRD: End-stage renal disease
CD20: B-lymphocyte antigen CD20
ISN/RPS: International Society of Nephrology/Renal Pathology Society
RAAS: Renin-angiotensin-aldosterone system
ACR: American College of Rheumatology
EULAR/ERA-EDTA: European League Against Rheumatism and European Renal Association-European Dialysis and Transplant Association
iNOS: Inducible nitric oxide synthase
Akt: Serine/threonine kinase 1
LPS: Lipopolysaccharide
MAPK: Mitogen-activated protein kinase
IFN-g: Interferon gamma
IL-2: Interleukin-2
ERK1/2: Extracellular signal-regulated kinases 1/2
CAT: Catalase
GSH: Glutathione
MDA: Malondialdehyde
PGE-2: Prostaglandin E-2
DOX: Doxorubicin
Smad: Small mothers against decapentaplegic
TGF-*β*1: Transforming growth factor beta 1
AA: Aristolochic acid
AAN: Aristolochic acid nephropathy
EGF: Epidermal growth factor
VEGF: Vascular endothelial growth factor
CTGF: Connective tissue growth factor
H-ras: Harvey rat sarcoma viral oncogene homolog
p53: Tumor protein p53
P450 3A4: Cytochrome P450 3A4
p-gp: P-glycoprotein 1
PPAR: Peroxisome proliferator-activated receptor
IL-17A: Interleukin-17A
IL-12: Interleukin-12
IL-4: Interleukin-4
IgG2: Immunoglobulin G2
FOXP3: Forkhead box P3
MCP-1: Monocyte chemoattractant protein-1
ROS: Reactive oxygen species
PUFA: Polyunsaturated fatty acid
MUFA: Monounsaturated fatty acid
ICAM-1: Intercellular adhesion molecule-1
GPx: Glutathione peroxidase
SOD: Superoxide dismutase
CTLA-4: Cytotoxic T-lymphocyte-associated protein 4
CCL-5: C-C motif chemokine ligand 5
CXCR3: C-X-C motif chemokine receptor 3
Nrf-2: Nuclear factor erythroid 2-related factor 2
HO-1: Heme oxygenase-1
MMP-3: Matrix metalloproteinase-3
eGFR: Estimated glomerular filtration rate
WfPB: Raw, whole-food, plant-based.
